# Flexural strength of dental adhesives with different photoinitiator systems

**DOI:** 10.4317/jced.61887

**Published:** 2024-08-01

**Authors:** Aldo-Pessoa de Figueiredo, Isaias-Donizeti Silva, Milton-Edson Miranda, Rafael-Pino Vitti, William-Cunha Brandt

**Affiliations:** 1 Faculdade São Leopoldo Mandic, Instituto de Pesquisas São Leopoldo Mandic, Campinas, SP, Brazil; 2 Department of Implantology, School of Dentistry, University Santo Amaro, São Paulo, SP, Brazil

## Abstract

**Background:**

The aim of this study was to investigate the flexural strength of dental adhesives containing different combinations of photoinitiators systems.

**Material and Methods:**

The organic matrix of the experimental adhesives was created using a blend of monomers: 50% by weight bisphenol-A glycidyl methacrylate (BisGMA) and 50% triethylene glycol dimethacrylate (TEGDMA). The photoinitiators utilized were camphorquinone (CQ) and phenylbis(2,4,6-trimethylbenzoyl)phosphine oxide (BAPO), with diphenyliodonium hexafluorophosphate (DPIHFP) and 2-(Dimethylamino)ethyl methacrylate (DMAEMA) as co-initiators. These photoinitiators and co-initiators were integrated into the organic matrix at a concentration of 0.5% by mass, resulting in the formation of 6 groups (n=12): CQ/DMAEMA (control); CQ/DMAEMA/DPIHFP; BAPO; BAPO/DMAEMA; BAPO/DPIHFP and BAPO/DMAEMA/DPIHFP. Samples measuring 7 mm in length, 2 mm in width, and 1 mm in height were prepared and subjected to a three-point flexural test. Data were analyzed using one-way ANOVA with Tukey’s post-hoc test (α=0.05).

**Results:**

Results indicated that groups incorporating BAPO and DPIHFP exhibited higher flexural strength compared to those with CQ and DMAEMA. The BAPO/DPIHFP group achieved the highest mean flexural strength values (*p*<0.001).

**Conclusions:**

These findings suggest that using adhesive systems with alternative photoinitiators and co-initiators can lead to superior flexural strength compared to conventional systems.

** Key words:**Photoinitiators, Dentin-bonding agents, Light-curing.

## Introduction

Currently, the dental market offers a wide variety of adhesive systems, which can be classified into conventional, self-etching, and universal systems (1-3). A common feature among these adhesive systems is the method of physical polymerization via blue light (430 to 480 nm). This is due to the presence of camphorquinone (CQ), a photoinitiator commonly found in resin composites, resin cements, and dental adhesives (4,5).

Camphorquinone (CQ) is a type II photoinitiator that becomes activated by blue light. For the polymerization process, type II photoinitiators require a co-initiator. Typically, a tertiary amine, such as dimethylaminoethyl methacrylate (DMAEMA), is used (4-6). Upon absorbing blue light, the CQ molecule becomes excited and interacts with DMAEMA, which donates electrons to form an ion pair. This interaction leads to the transfer of a proton, generating free radicals. The resulting amino radical then initiates the polymerization process (5,6).

Photoinitiators must exhibit sufficient photochemical and photophysical properties to effectively trigger the reaction process. Ideal characteristics include ease of manipulation, solubility in the reaction medium, high molar extinction coefficient, biocompatibility, and a high quantum yield for generating active species (free radicals (7). Additionally, they should exhibit low chemical degradation and not cause yellowing. However, these last two criteria present challenges for CQ-based resin materials. CQ is a yellowish solid compound containing a chromophoric group in its molecular structure, which in large amounts can lead to unwanted yellowing and affect the final appearance of the polymerized material (6,7). Moreover, CQ and DMAEMA are prone to chemical degradation even after polymerization, and this yellowish discoloration can pose a problem for color matching. The discoloration is primarily due to DMAEMA, which undergoes oxidation over time, causing the dental resin to change color (5,7).

To address these issues, various photoinitiators have been developed and incorporated into resin materials to act either independently (type I) or synergistically (type II) (4-7). Type I photoinitiators generate free radicals through the fragmentation of the photoinitiator molecule itself. Compounds like phenylbis(2,4,6-trimethylbenzoyl)phosphine oxide (BAPO) and α-diketones are type I photoinitiators commonly used in resin formulations to enhance polymerization kinetics and reduce yellowing effects. These photoinitiators are typically white or lightly yellow with low saturation and can exist as solids (powder) or liquids at room temperature. Unlike CQ, type I photoinitiators absorb light within the lower energy spectrum, specifically between 350 and 430 nm (6-8).

Using co-initiators other than tertiary amines in resin-based materials aims to enhance the kinetics of the polymerization reaction and reduce post-polymerization degradation (9). Typically, iodonium salts operate through a ternary reaction system (photoinitiator + tertiary amine + diphenyliodonium hexafluorophosphate - DPIHFP), which improves the color stability of resin materials, reduces yellowing, and increases the depth of polymerization. Consequently, DPIHFP can facilitate the polymerization reaction in conditions with limited light availability (10).

Optimal photoactivation is achieved when monomers are fully converted into polymer. However, in practice, the degree of conversion is never complete. One way to improve monomer conversion is by increasing the energy density (time and irradiance) during light-curing (4,11). However, extending the light-curing time can be clinically inconvenient and may lead to issues such as increased temperature. Additionally, more energy does not always result in more free radical generation. Therefore, modifying the photoinitiator system is crucial for reducing residual monomers and enhancing the degree of conversion (5,11).

This study aimed to evaluate the flexural strength of experimental resin-based adhesives containing various combinations and concentrations of photoinitiator systems (CQ and BAPO) and co-initiators (DMAEMA and DPIHFP). The hypothesis was that the adhesive system containing BAPO and DPIHFP would exhibit the highest flexural strength values.

## Material and Methods

-Experimental design

In this *in vitro* study, the factors investigated included various combinations and concentrations of photoinitiator and co-initiator systems at six levels: CQ/DMAEMA (control), CQ/DMAEMA/DPIHFP, BAPO, BAPO/DMAEMA, BAPO/DPIHFP, and BAPO/DMAEMA/DPIHFP. The response variables were quantitatively assessed for flexural strength (n=12). The present study was submitted to the Ethics Committee of São Leopoldo Mandic (approval number #2015/0430) and was exempted from review because it involved solely laboratory research without any involvement of human subjects or biological materials.

-Formulation of experimental adhesive systems 

Experimental formulations of resin-based adhesive systems were prepared using a 50/50 weight mixture of bisphenol-A glycidyl methacrylate (BisGMA; Sigma-Aldrich, St. Louis, MO, USA) and triethylene glycol dimethacrylate (TEGDMA; Sigma-Aldrich). The monomers were blended for 40 seconds at 2400 rpm using a centrifugal mixing device (SpeedMixer, DAC 150.1 FVZ-K, Hauschild Engineering, Hamm, Germany). Photoinitiators CQ (Sigma-Aldrich) or BAPO (Sigma-Aldrich) were added to the monomer blend, along with co-initiators DMAEMA (Sigma-Aldrich) and/or DPIHFP (Sigma-Aldrich) in a 1:1 weight ratio. These initiator and co-initiators systems were incorporated into the monomer matrix at a concentration of 0.5% by weight, along with 0.1% by weight of an inhibitor (butylated hydroxytoluene - BHT; Sigma-Aldrich). The mixture was centrifuged for 1 minute at 2000 rpm under an 80-mmHg vacuum. The manipulation of the experimental adhesives was carried out under orange filtered light.

The resins were stored in plastic containers wrapped in light-blocking paper. The allocation of materials in the containers was not disclosed to the operators responsible for the flexural strength testing (A. P. F.) and the statistical analysis (W. C. B.), ensuring that these two analyses remained blinded.

-Flexural strength

**Figure 1:**
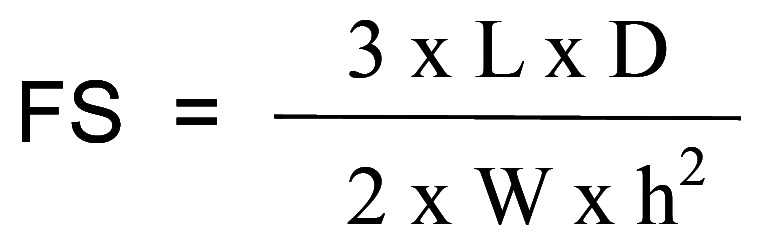
Equation.

The flexural strength of the experimental adhesives was assessed using the three-point bending test. Seventy-two bar-shaped specimens were created using a silicone mold (Elite, Zhermack, Badia Polesine, Italy) measuring 7 mm in length, 2 mm in width, and 1 mm in height. The experimental adhesives were applied to the molds, and a strip of polyester matrix was placed over each sample. Subsequently, the experimental adhesives were light-cured (Valo®, Ultradent, South Jordan, USA) for 20 seconds with the light tip in contact with the polyester strip, delivering an irradiance of 1,200 mW/cm2 (24 J/cm2). The light-curing unit tip, approximately 10 mm wide, covered the entire sample surface. After being stored dry for 14 days at 37ºC, the specimens underwent the three-point flexural test using a universal testing machine (EMIC, model DL 2000, São José dos Pinhais, PR, Brazil) at a crosshead speed of 1.0 mm/min with a 10 Kgf load cell until failure. The support used for specimen support consisted of two metal bars positioned 4.20 mm apart, attached to an acrylic base. Flexural strength (FS) was expressed in megapascals (MPa) using the following equation: (Fig. 1).

where L was the maximum load at failure (N), D was the distance (span) between the rods, W was the specimen’s width, and h was the specimen height.

-Statistical analysis

The statistical analysis included preliminary tests to assess the normality of the sample distribution. Upon confirming normality (*p*>0.05 in the Shapiro-Wilk test) and homoscedasticity (*p*>0.05 in the Levene test), parametric statistical test was employed. One-way analysis of variance (ANOVA) with Tukey’s post-hoc test (α=0.05). Statistical analysis was conducted using SigmaPlot 14.0 (Systat Software Inc., San Jose, California, EUA).

## Results

 Table 1 displays the mean values and standard deviations of the flexural strength of the experimental adhesives.

The results indicate that the flexural strength of the experimental adhesives was significantly influenced by the initiators and co-initiators (*p*<0.001). Specifically, the lowest mean flexural strength was observed in the CQ/DMAEMA (control) group ( Table 1). Conversely, the highest mean FS was observed in the BAPO/DPIHFP group (*p*<0.001), which did not statistically differ from the BAPO/DMAEMA and BAPO/DMAEMA/DPIHFP groups (*p*>0.05).

Discussion 

The hypothesis was confirmed as the experimental adhesives containing BAPO and DPIHFP demonstrated the highest flexural strength values ( Table 1). BAPO is a photoinitiator known for its high reactivity, as one BAPO molecule can generate four free radicals. This molecule contains two calcium-phosphorus (C-P) bonds, which are broken by photons generated during photoactivation, initiating polymerization (7,12,13). Consequently, there are always two potentially active free radicals per absorbed photon, resulting in a higher molar extinction coefficient compared to CQ. The increased quantity and availability of free radicals generated by BAPO facilitate the onset of polymerization reaction, consequently enhancing the material’s physical and mechanical Properties (7,13), such as flexural strength (Table 1). This explains why the experimental adhesive containing only BAPO, without a co-initiator, outperformed the control group (CQ/DMAEMA).

BAPO (a type-1 photoinitiator) is a white powder with light absorption near 370 nm, which is a different wavelength range from that required for CQ photoactivation. This necessitates the use of a light source capable of producing a broader spectral range (polywave) when BAPO is present in the resin material composition. The light curing unit utilized in this study (Valo®, Ultradent) is an LED system with a wavelength range between 395 and 480 nm required for generating free radicals through the photofragmentation of its molecule (4-7,12,13).

CQ is an alpha-diketone and serves as a type-2 photoinitiator. This photoinitiator exhibits an absorbance maximum peak at 468 nm. As CQ can dissolve in various resins like TEGDMA, it displays a phenomenon called solvatochromic shift. The initiation mechanism of CQ involves the carbonyl group. This group, along with DMAEMA, enters an excitatory state (triplet state) upon absorption of blue light (468 nm). The ionic pair formed by DMAEMA after interaction with CQ generates free radicals, which are energetic molecules responsible for initiating the polymerization process (4-7).

However, in the polymerization process of CQ/DMAEMA, there is a slow hydrogen abstraction, which could limit the concentration of free radicals (5-7), leading to a decrease in flexural strength values (Table 1). Additionally, the time required to form triplet exciplex is limited, as the half-life of CQ triplet is approximately 0.05 s (14). Beyond this time, the CQ triplet disintegrates to its basic state, halting the production of free radicals. Another issue is that this system consists of two components, and their interaction is influenced by the viscosity of the medium. In low-viscosity formulations, the reduction of the CQ triplet and amines is closely associated with the diffusion reaction of these reagents. Conversely, in high-viscosity environments, bimolecular systems are constrained in their reactivity due to diffusion-controlled processes (6,15).

In essence, the process of radical generation through photophysical and photochemical means can be categorized into two primary mechanisms: (i) photo-scission, typical of type I photoinitiators, and (ii) hydrogen abstraction, which necessitates a co-initiator, typical of type II photoinitiators. When a type I photoinitiator is exposed to ultraviolet light, it undergoes homolytic cleavage processes upon reaching excited singlet and triplet states through intersystem crossing. This cleavage often occurs at bonds like C-C bonds adjacent to carbonyl groups (α-cleavage), resulting in the formation of two radicals via the Norrish type I reaction (15,16). Conversely, radical generation with a type II system is more intricate and involves three steps: (i) intermolecular exciplex formation once the photoinitiator reaches an excited triplet state, (ii) rapid electron transfer, and (iii) gradual proton transfer. It’s important to note that type II systems are generally less efficient due to bimolecular processes, back electron transfer, and the solvent cage effect in aqueous solutions (15).

Another noteworthy point is that CQ is considered toxic (17,18), as it has the potential to alter the metabolism of structural lipids, thereby affecting membrane integrity and permeability. Although CQ is less cytotoxic than BAPO, it possesses genetic toxicity potential due to the production of Reactive Oxygen Species (ROS) and Reactive Nitrogen Species (RNS) (19). These clinical concerns have led to the consideration of other photoinitiators for the production of commercial composites. As an alternative, DPIHFP has been added as an initiator (18).

DPIHFP functions as an effective photoinitiator in cationic polymerization; however, it only becomes active when irradiated with a source of ultraviolet light (18). The strategy for using it in the biological field involves employing a photosensitizer that absorbs in the visible light spectrum and can react with the iodonium salt, which acts as a co-initiator by promoting its decomposition. Consequently, it becomes possible for the iodonium salts to also participate in the polymerization of methacrylate radicals (18,20). The proposed mechanism of action for adding this component to adhesives is that after the exciplex formation between DPIHFP and CQ, the reaction of this complex with the tertiary amine regenerates iodonium salts and reacts with unreacted CQ molecules, forming new exciplex states, thereby serving as an additional source of radicals to initiate polymerization (20).

Based on the results presented in Table 1, it was observed that in the flexural strength test, the experimental adhesives containing DPIHFP showed equal or superior results compared to formulations without this substance. DPIHFP acts with the CQ/amine system through the abstraction and formation of phenyl radicals. Additionally, this compound synergistically interacts with other photoinitiators, as observed in the case of BAPO (18,20,21), which was confirmed in this study. When in contact with water or solvents, DPIHFP increases the conversion rate of the CQ/amine system compared to its absence, favoring adhesive formulations containing this salt. DPIHP has been reported to enhance the polymerization rate and degree of conversion of resin-based materials (18,21). This enhancement may be attributed to a more reactive phenyl group resulting from the reaction between DPIHP and the amine (19,21).

The findings of this study demonstrated that BAPO and DPIHFP leads to improved flexural strength compared to conventional adhesives employing CQ and DMAEMA. This presents a viable option for either substituting or complementing the conventional system in BisGMA/TEDGMA-based materials. Further exploration into the polymerization mechanism and its impact on the polymeric network is warranted to inform future research directions. Additionally, future studies should assess the chemical, biocompatibility, and other physico-mechanical properties of experimental resin-based adhesives.

## Conclusions

The experimental adhesives containing the BAPO (photoinitiator) and DPIHFP (co-initiator) system exhibited higher flexural strength values compared to those demonstrated by the conventional system containing CQ and DMAEMA.

## Figures and Tables

**Table 1:** Mean and standard deviation (±SD) for flexural strength (MPa) values.

Groups	Flexural strength
CQ/DMAEMA (control)	72.64 (13.37) c
CQ/DMAEMA/DPIHFP	121.51 (21.22) b
BAPO	110.39 (26.03) b
BAPO/DMAEMA	135.48 (22.86) ab
BAPO/DPIHFP	150.54 (21.05) a
BAPO/DMAEMA/DPIHFP	133.13 (31.26) ab

Different letters indicate statistically significant diferences for comparison between experimental adhesives (column) (*p*<0.05).

## Data Availability

The datasets used and/or analyzed during the current study are available from the corresponding author.
